# Interferon α2–Thymosin α1 Fusion Protein (IFNα2–Tα1): A Genetically Engineered Fusion Protein with Enhanced Anticancer and Antiviral Effect

**DOI:** 10.3390/ma14123318

**Published:** 2021-06-15

**Authors:** Muhammad Shahbaz Aslam, Syed Zohaib Javaid Zaidi, Rabail Hassan Toor, Iram Gull, Muhammad Mudassir Iqbal, Zaigham Abbas, Imran Tipu, Aftab Ahmed, Muhammad Amin Athar, Christian Harito, Sammer-ul Hassan

**Affiliations:** 1 Institute of Biochemistry and Biotechnology, University of the Punjab, Quaid-i-Azam Campus, Lahore 54590, Pakistan; iram.ibb@pu.edu.pk (I.G.); mudassiribb@gmail.com (M.M.I.); amatmian@yahoo.com (M.A.A.); 2 Institute of Chemical Engineering and Technology, University of the Punjab, Lahore 54590, Pakistan; 3 School of Biological Sciences, University of the Punjab, Quaid-i-Azam Campus, Lahore 54590, Pakistan; rabail.toor@yahoo.com (R.H.T.); aftab.nays@gmail.com (A.A.); 4 Department of Microbiology & Molecular Genetics, University of the Punjab, Quaid-i-Azam Campus, Lahore 54590, Pakistan; zaigham.mmg@pu.edu.pk; 5 Department of Life Sciences, University of Management & Technology, Lahore 54770, Pakistan; imran.tipu1@gmail.com; 6 Industrial Engineering Department, Faculty of Engineering, Bina Nusantara University, Jakarta 11480, Indonesia; christian.harito@binus.ac.id; 7 Bioengineering Research Group, Faculty of Engineering and Physical Sciences, University of Southampton, Southampton SO17 1BJ, UK

**Keywords:** fusion protein, anti-proliferative effect, antiviral effect, genotoxic effect, relative expression

## Abstract

Human interferon α2 (IFNα2) and thymosin α1 (Tα1) are therapeutic proteins used for the treatment of viral infections and different types of cancer. Both IFNα2 and Tα1 show a synergic effect in their activities when used in combination. Furthermore, the therapeutic fusion proteins produced through the genetic fusion of two genes can exhibit several therapeutic functions in one molecule. In this study, we determined the anticancer and antiviral effect of human interferon α2–thymosin α1 fusion protein (IFNα2–Tα1) produced in our laboratory for the first time. The cytotoxic and genotoxic effect of IFNα2–Tα1 was evaluated in HepG2 and MDA-MB-231 cells. The in vitro assays confirmed that IFNα2–Tα1 inhibited the growth of cells more effectively than IFNα2 alone and showed an elevated genotoxic effect. The expression of proapoptotic genes was also significantly enhanced in IFNα2–Tα1-treated cells compared to IFNα2-treated cells. Furthermore, the HCV RNA level was significantly reduced in IFNα2–Tα1-treated HCV-infected Huh7 cells compared to IFNα2-treated cells. The quantitative PCR analysis showed that the expression of various genes, the products of which inhibit HCV replication, was significantly enhanced in IFNα2–Tα1-treated cells compared to IFNα2-treated cells. Our findings demonstrate that IFNα2–Tα1 is more effective than single IFNα2 as an anticancer and antiviral agent.

## 1. Introduction

Interferon-alpha 2 (IFNα2) is a type of biopharmaceutical which is equally effective for the treatment of viral infections and different types of cancers [1]. It directly acts on tumor cells or exerts its effect indirectly through immune cells. IFNα2 regulates the expression of multiple interferon regulatory genes (IRGs), and effector proteins of these IRGs directly affect proliferation, differentiation, growth and other functions of tumor cells. Ultimately, IFNα2 exerts its anti-proliferative effect on cancerous cells through apoptosis, cell cycle arrest, anti-angiogenesis or immune system activation [2]. IFNα2 induces the expression of other cytokines and activates immune cells such as natural killer (NK) cells, macrophages, dendritic cells (DCs), and cytotoxic T cells to regulate immune response, which further generates anti-tumor immunity [3,4]. IFNα2 inhibits viral infection through the induction of interferon stimulated genes (ISGs), the products of which inhibit the replication of viruses by acting at different stages of the virus life cycle or through the activation of immune cells such as NK cells, as in the case of hepatitis C virus infection, the clearance of the virus is linked with the activation of an immune response to the virus [5,6,7,8]. Although IFNα2 performs most of its activities through the activation of JAK-STAT (Janus Kinase/Signal Transducer and Activator of Transcription) signaling pathways, studies show that it can also activate other signaling pathways such as MAP kinase signaling pathways [9,10] and the PI3K-AKT-MTOR (Phosphoinositide 3-Kinase/Protein Kinase B/Mammalian Target of Rapamycin) signaling pathway [11,12].

Thymosin alpha 1 (Tα1), a peptide of 28 amino acids, is therapeutically used for chronic HBV (hepatitis B virus) and HCV (hepatitis C virus) infections and different types of cancers through its immune-modulating and direct acting effect on tumor or infected cells [13]. It acts through Toll-like receptors (TLRs) [14,15] to modulate immune response, which is essential for fighting against viral infections, which impair immune response and different types of cancers as depressed cellular immunity leads to an enhanced progression of cancer [16]. Tα1 also activates other signaling pathways shared by other cytokines, which shows synergy between Tα1 and other cytokines [17]. Through its immune-modulating effect, it increases the activity of NK cells to mediate NK cell-mediated cytotoxicity of cells infected with virus and tumor cells. Tα1 increases the level of cytotoxic T cells, T helper cells, DCs and macrophages which further show antiviral and anti-tumor effect [18,19]. Interestingly, Tα1 also prevents the inflammatory cytokine storm of immune response by increasing the production of regulatory T cells [20]. In addition, it also increases the expression of marker proteins on the surface of virus-infected cells and tumor-specific antigens on tumor cells to prevent the escape of these cells from recognition by immune cells [20,21,22]. Due to a wide range of therapeutic applications, it is being used either alone or in combination with other therapeutics [23,24,25]. Studies show that the use of Tα1 in combination with IFNα or IL2 gives a higher biological effect [26] in the treatment of viral infections and cancer.

According to estimates by the World Health Organization (WHO), viral hepatitis B and C infect about 325 million people globally, which leads to ~1.4 million deaths in one year [27,28]. Cancer is also widely reported as a major cause of deaths, and its incidence and mortality rate are increasing rapidly worldwide [29]. Multiple studies show that the therapeutic use of IFNα2 with Tα1 in combination is more effective and safer for chronic hepatitis infections as well as various types of cancer due to synergy in their activities [17,30]. In addition, the recombinant therapeutics produced by the genetic fusion of different genes may exhibit several functions in a single molecule [17,31,32,33]. Therefore, a single fusion protein constructed by the genetic fusion of two genes can be used as a substitute for two proteins that are used in combination for therapy.

In light of the above, the anticancer and antiviral activities of IFNα2–Tα1 constructed by the fusion of IFNα2 and Tα1 genes in our laboratory were determined. Here, we report for the first time that IFNα2–Tα1 inhibits the growth of HepG2 and MDA-MB 231 cells more effectively than IFNα2 alone. Simultaneously, IFNα2–Tα1 also inhibited the replication of HCV in Huh7 cells more effectively than reference IFNα2. The anti-proliferative effect of IFNα2–Tα1 was determined by neutral red and MTT (3-4,5-dimethylthiazol-2-yl-2,5-diphenyl tetrazolium bromide) assay, while its genotoxic effect was determined by comet assay. The antiviral effect of IFNα2–Tα1 was determined by in vitro anti-HCV assay in HCV-infected Huh7 cells. Furthermore, the change in the expression of multiple genes related to anticancer, antiviral and immunomodulatory effects was measured by quantitative real-time polymerase chain reaction (qPCR) in IFNα2–Tα1-treated cells and compared with IFNα2-treated cells. 

## 2. Materials and Methods

### 2.1. Materials

The human HepG2 and MDA-MB-231 cells were maintained at the cell culture laboratory of the School of Biological Sciences (SBS), while the human liver cell line (Huh7) was maintained in the cell culture laboratory of CEMB (University of the Punjab, Lahore, Pakistan) in complete DMEM medium (Gibco, ThermoFisher Scientific, Carlsbad, CA, USA). The culture media (DMEM; cat# 11965092), FBS (cat# 16000044), penicillin–streptomycin (cat#15140122), trypsin (cat# 25200056), MTT (cat# M6494), TRIzol reagent (cat # 15596018), cDNA synthesis kit (cat# K1622) and qPCR master mix (K0222) were purchased from Thermo Fisher Scientific (Carlsbad, CA, USA). Crystal violet solution (cat# V5265) and neutral red (cat# N4638) were purchased from Sigma Aldrich (now Merck, KGaA, Dramstadt, Germany). Artus^®^ HCV RG RT-PCR kit (cat# 4518265; Qiagen, Hilden, Germany) was kindly provided by Decent Hormone Lab, Lahore, Pakistan. All other analytical grade chemicals and reagents were used.

### 2.2. Methods

#### 2.2.1. Cell Attachment Assay

A total of 200 µL of IFNα2–Tα1 (1 and 10 ng/mL), IFNα2 (1 and 10 ng/mL) and 1X PBS as control were added individually in a 96-well microtiter plate. The plate was incubated at 4 °C overnight and further blocked with 2% BSA in DMEM medium for 2 h at 37 °C. HepG2 cells (30,000/well) were added in a pre-coated plate and incubated for 1 h at standard conditions. The plate was washed thrice with 1X PBS to remove unbound cells, and bound cells were fixed. The cells were photographed after staining for 10 min in crystal violet solution (0.5%). Then, the plate was washed thrice with 1X PBS, and crystal violet was extracted from wells by 10% acetic acid. The absorbance of adhered HepG2 cells after extraction was measured by ELISA reader (ELx808 BioTek Instruments, Winooski, VT, USA) at 510 nm. The formula (AT–AC)/AC × 100% was used to calculate the relative adhesion of cells (%). The AT is the absorbance of test protein-treated cells, while AC is the absorbance of cells treated with the control [34].

#### 2.2.2. Anti-Proliferative Effect

The anti-proliferative effect of IFNα2–Tα1 in HepG2 and MDA-MB-231 cells was examined by neutral red [35] and MTT [36] assay in comparison with reference IFNα2, while the comet assay was used to determine the genotoxic effect.

##### Neutral Red Assay

Briefly, HepG2 and MDA-MB-231 cells were seeded in triplicate at a density of 10,000/cm^2^ with the medium in a 96-well plate and grown for 24 h at standard conditions. The cells were then treated with various concentrations (1–10 ng/mL) of IFNα2–Tα1 and IFNα2 diluted in a fresh medium for 24 h. The medium was aspirated, and cells were incubated with neutral red dye (40 µg/mL) at 37 °C for 2 h in a 5% CO_2_ atmosphere. The working solution of neutral red was prepared by diluting the stock solution (4 mg/mL in 1X PBS) of neutral red in a ratio of 1:100 with fresh medium to a final concentration of 40 µg/mL. After 2 h, the neutral red solution was aspirated, and cells were then washed with 1X PBS thrice. The images of cells were taken by an inverted microscope, and cells were destained with 200 µL of neutral red de-staining solution for 10 min. The OD of the supernatant was measured at 570 nm wavelength. Average values were calculated, and the graph was plotted for % age cell viability against each concentration of both protein samples for both types of cells. The culture medium with 1X PBS was used as a positive control for the cells.

##### MTT Assay

As described earlier in the neutral red assay, the HepG2 and MDA-MB231 cells were treated with various (1–10 ng/mL) concentrations of IFNα2–Tα1 and reference IFNα2 with the medium in a 96-well plate for 24 h. After treatment, 10 µL of MTT solutions (5 mg/mL) was added to each well in a complete medium and incubated at 37 °C for 4 h in a 5% CO_2_ atmosphere. The medium was removed, and cells were then washed with 1X PBS. After that, 150 µL of acidified isopropanol was added to the cells and incubated for 10 min at room temperature. The absorbance was measured at 570 nm, and average values were taken. The graph was plotted for percentage cytotoxicity against each concentration of both proteins for both cell types. The % cytotoxicity by different concentrations of sample and reference protein compared to the cells without treatment was determined with the formula (% cytotoxicity = cells absorbance with no treatment—cells absorbance with treatment/cells absorbance with no treatment).

#### 2.2.3. Genotoxic Effect Analysis by Comet Assay

HepG2 cells and MDA-MB231 cells (10,000/well) containing DMEM complete medium were incubated separately in a CO2 incubator at 37 °C for 24 h in a 6-well culture plate. The next day, the medium was aspirated, and a fresh medium containing 10 ng/mL of each IFNα2–Tα1 and IFNα2 was added and the plate was incubated at standard conditions for 24 h. The control was prepared with 1X PBS without any protein. The control and treated cells were trypsinized and counted on a hemocytometer. The cells (5 × 10^5^/mL) were suspended in 1X PBS and analyzed for comet formation. Briefly, 300 µL of 0.5% agarose in 1X TAE was spread on the microscope slides, and slides were solidified at room temperature. Then, 100 µL (10,000 cells) of both treated and control cells was mixed with 100 µL of 0.5% low melting agarose and layered on agarose coated slides. The slides were allowed to solidify and then dipped in lysis solution “(100 mM Tris-Cl pH 10, 0.1 M EDTA, 2.5 M NaCl, 1% sodium sarcosinate in 1% Triton X-100 and 10% DMSO)” for 1 h at 4 °C. After 20 min, electrophoresis was conducted for 20 min at 25 volts in an electrophoresis tank using running buffer (0.001 M EDTA, 0.3 M NaOH, pH 8.5). The slides were washed with 1X PBS thrice and immersed in a neutralizing buffer (0.4 M Tris-Cl buffer, pH 7.5) for 10 min. The slides were stained in 20 µg/mL ethidium bromide solution and analyzed with a fluorescence microscope (Nikon Eclipse TS-100, Nikon Instruments Inc., Melville, NY, USA). Cometscore 2.0 was used to analyze the comets. Multiple comets were selected for each slide, and eight different parameters (comet length, comet height, head diameter, head area, percentage DNA in head, tail length, tail area and percentage DNA in tail) were studied for each comet [37]. The average of values for each parameter was taken, and the graph was plotted to compare the genotoxic effect of IFNα2–Tα1 with the reference IFNα2.

#### 2.2.4. Antiviral Activity

The antiviral activity of IFNα2–Tα1 was determined by in vitro anti-HCV assay in HCV-infected Huh7 cells.

##### In Vitro Toxicity Analysis

The toxicity of IFNα2–Tα1 in Huh7 cells was determined by the trypan blue exclusion test. Huh7 cells (10,000/well) were added in each well of a 48-well culture plate and treated with medium containing various concentrations of IFNα2–Tα1 (0.2–2 ng/mL) for 24 h in an incubator at standard conditions. Culture medium with 1X PBS was used as a control. The cells were trypsinized after incubation and counted by a hemocytometer. The concentration of IFNα2–Tα1 at which ~85% cells were viable was considered as non-toxic for cells. 

##### In Vitro Anti-HCV Assay

Huh7 cells (10,000/well) were seeded in 6-well cell culture plates and grown in DMEM medium for 24 h at standard conditions. Next day, the cells were infected with HCV (genotype 3a) positive serum. After infection, the cells were treated with a medium containing various concentrations (0.2–1 ng/mL) of IFNα2–Tα1 and reference IFNα2 for 24 h at 37 °C. Next day, total RNA was extracted from infected and untreated infected cells by Trizol reagent. The HCV RNA was quantified by quantitative PCR using Artus^®^ HCV RG RT-PCR kit following the manufacturer’s instructions. The HCV RNA in cells treated with various concentrations of sample proteins was compared with the control cells (untreated infected cells). The experiments were performed in triplicate.

#### 2.2.5. Expression Analysis

The change in expression of genes associated with anticancer, antiviral and immunomodulatory effects in IFNα2–Tα1 and IFNα2-treated cells was quantified by quantitative PCR and compared with untreated control cells. TRIzol reagent was used for the extraction of total RNA from treated and untreated cells. The RNA was quantified by NanoDropTM spectrophotometer and cDNA was prepared using cDNA synthesis kit from 1 µg of RNA. Oligonucleotide primers designed by software “primer 3 plus” and synthesized commercially were used for quantification of the mRNA level of genes. CFX96 Real-time PCR Detection System (Bio-Rad Laboratories, Hercules, CA, USA) was used to quantify the expression of different genes in treated and untreated cells in triplicate. The expression of the GAPDH gene was used as an internal control to normalize the expression of target genes. The control sample was used to calibrate the relative expression of target genes. The reaction mixture was prepared according to instructions given in the kit manual (Thermo Fisher Scientific, Carlsbad, CA, USA). The thermal cycling protocol was set as initial denaturation at 95 °C for 3 min followed by 45 cycles of denaturation at 95 °C for 20 s, annealing and extension at 60 °C for 20 s. The specificity of quantitative PCR assay was determined by the melt curve analysis. The data were analyzed with CFX Manager Software (Bio-Rad, Hercules, CA, USA).

#### 2.2.6. Statistical Analysis

All the experiments in this study were performed in triplicate and the results were statistically analyzed by calculating the Mean ± Standard Deviation (SD). Statistical analysis was performed using IBM SPSS statistics 20 (SPSS 20) and GraphPad Prism (v 7.03). The difference among three groups was considered significant for a value of *p* < 0.05 with 95% confidence.

## 3. Results

### 3.1. In Vitro HepG2 Cells Attachment Assay

**Figure 1:**
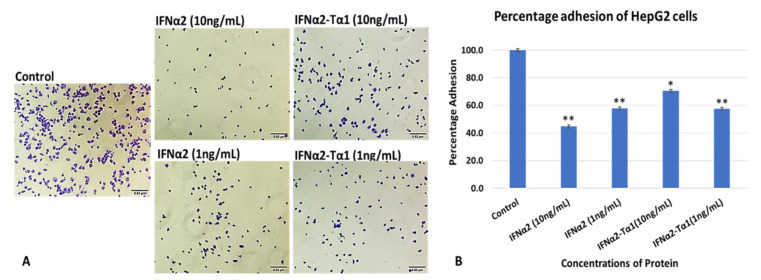
(**A**) Cell attachment assay of IFNα2–Tα1 to HepG2 cells in comparison with reference IFNα2 and control (PBS). (**B**) Crystal violet assay to determine percentage adhesion of IFNα2–Tα1 to HepG2 cells in comparison with reference IFNα2 at different concentrations. The experiment was performed in triplicate. “Data are represented as Mean ± SD of technical and biological replicates. For statistical analyses, One-Way ANOVA with Dunnett’s test for multiple comparison was performed using GraphPad Prism (v 7.03) (*p* ≤ 0.05; ns = not significant, * *p* ≤ 0.05, ** *p* ≤ 0.01).

The cell attachment ability of IFNα2–Tα1 to HepG2 cells was compared with IFNα2. The cells bound to microtiter plate wells were stained with crystal violet and photographed (Figure 1A). The relative adhesion of cells (%) was measured by ELISA reader at 595 nm (Figure 1B). We observed that IFNα2–Tα1 showed higher attachment ability to HepG2 cells (70.3%), especially at 10 ng/mL concentration (Figure 1A,B) as compared to IFNα2 (44.7%) at similar concentration. The percentages were calculated in comparison to the negative control.

### 3.2. Enhanced Inhibition of HepG2 and MDA-MB-231 Cell Proliferation by IFNα2–Tα1

**Figure 2:**
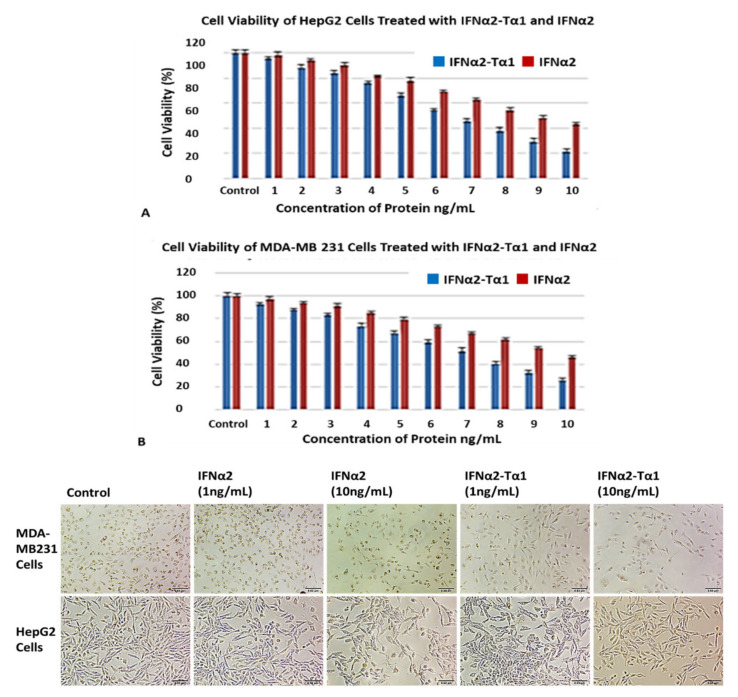
Inhibition of HepG2 and MDA-MB-231 cell proliferation by IFNα2–Tα1 or reference IFNα-2. (**A**) HepG2 cells and (**B**) MDA-MB-231 cells treated with various concentrations of IFNα2–Tα1 or IFNα2 alone for 24 h and cell viability (%) determined by neutral red assay. Data are represented as Mean ± SD (n = 3, two-way ANOVA, *p* < 0.05 considered significant with 95% confidence). (**C**) IFNα2–Tα1 and IFNα2-treated MDA-MB-231 and HepG2 cells observed under 10× magnification after staining with neutral red dye.

We determined the anti-proliferative effect of IFNα2–Tα1 by neutral red and MTT assay in HepG2 and MDA-MB-231 cells and compared it with the effect of reference IFN-α2. The cells were treated with different concentrations of IFNα2–Tα1 or IFNα2 for 24 h and the effect of proteins on the growth of cells was measured by both neutral red and MTT assay. The assays were repeated in triplicate and the cell viability (%) of IFNα2–Tα1-treated cells was compared with IFN-α2-treated cells at the same conditions. We observed that IFNα2–Tα1 showed significantly higher anti-proliferative effect as compared to IFNα2 alone at all concentrations in both HepG2 and MDA-MB-231 cells. For example, IFNα2–Tα1 at a concentration of 10 ng/mL showed 78.77% (Figure 2A) and 73.92% (Figure 2B) percentage inhibition of HepG2 and MDA-MB 231 cells respectively compared to control cells, whereas treatment with IFNα2 alone at same concentration showed 57.11% (Figure 2A) and 53.76% (Figure 2B) inhibition in cell proliferation respectively compared to control cells, as determined by neutral red assay.

**Figure 3:**
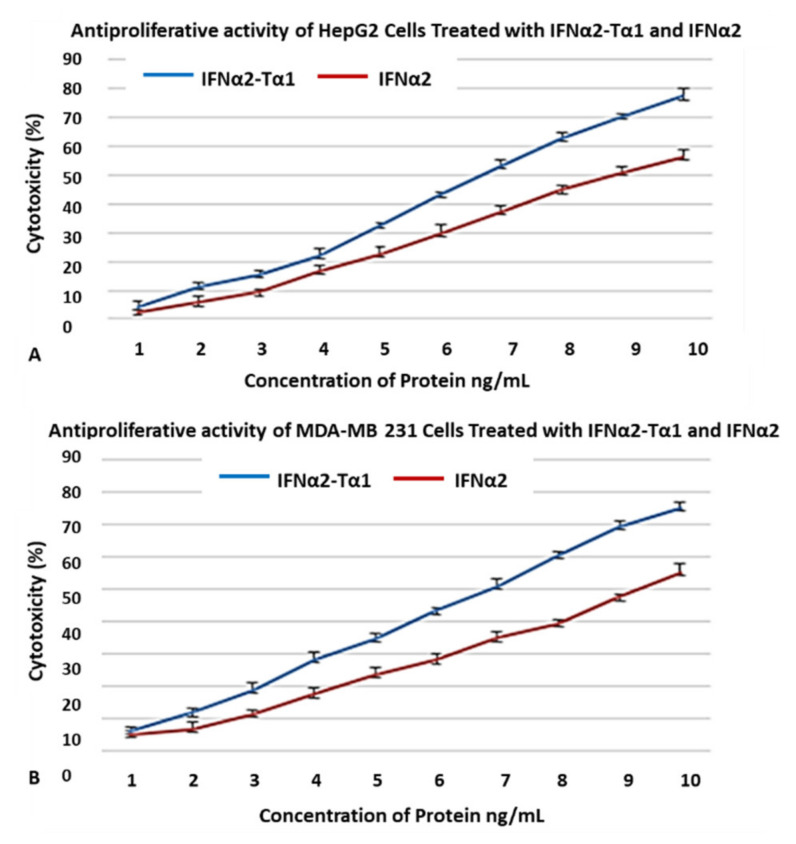
Anti-proliferative effect of IFNα2–Tα1 or IFNα2 alone in HepG2 and MDA-MB-231 cells measured by MTT assay. (**A**) HepG2 cells and (**B**) MDA-MB-231 cells treated with various concentrations of IFNα2–Tα1 or IFNα2 for 24 h and analysis of cell growth inhibition by MTT assay. Data are represented as Mean ± SD (n = 3, two-way ANOVA, *p* < 0.05 considered significant with 95% confidence).

Similarly, in the case of MTT assay, it was observed that the viability (%) of HepG2 and MDA-MB-231 cells was decreased equal to 77.32% (Figure 3A) and 75.11% (Figure 3B) respectively in response to treatment with 10ng/ml IFNα2–Tα1 compared to control cells whereas we observed 56.23% (Figure 3A) and 54.98% (Figure 3B) decrease in % viability of cells respectively, in response to treatment with reference IFN-α2 at a concentration of 10 ng/mL. Collectively the results indicated IFNα2–Tα1 is more efficacious in inhibiting growth of cancerous cells compared to IFNα2 alone with about 20% more inhibitory activity on proliferation of HepG2 and MDA-MB231 cells. These results further confirmed that the fusion of Tα1 with IFN-α2 enhanced the anti-proliferative effect of IFN-α2 against both liver and breast cancer cells.

### 3.3. Genotoxic Effect of IFNα2–Tα1 in HepG2 Cells and MDA-MB-231 Cells

**Figure 4:**
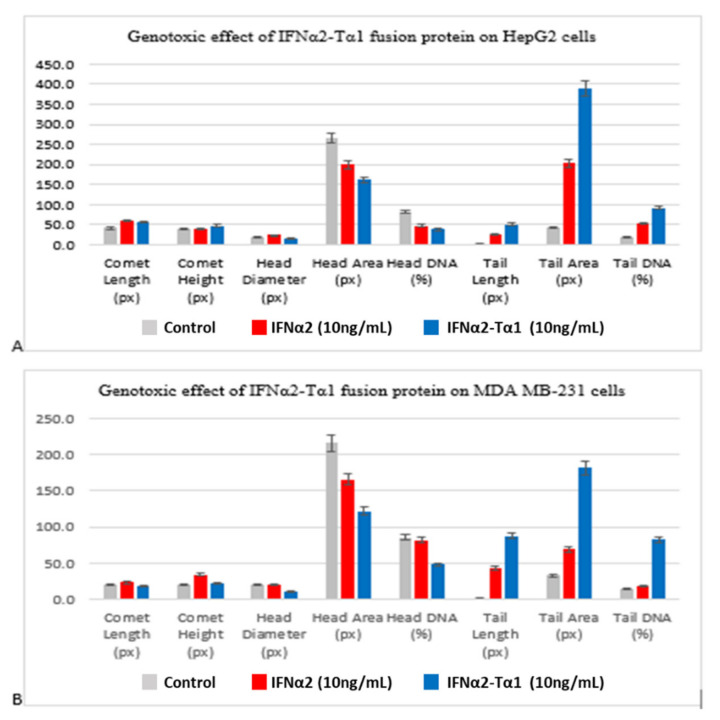
Comparison of eight different comet parameters (comet length, comet height, head diameter, head area, head DNA (%), tail length, tail area and tail DNA (%)) of IFNα2–Tα1, IFNα2 and control HepG2 cells (**A**) and MDA-MB-231 cells (**B**). The graphs clearly show that for both cell lines, tail length, tail area and % tail DNA are higher in IFNα2–Tα1-treated cells in comparison with IFNα2 and control cells, indicating more DNA damage in fusion protein-treated cells. Data are represented as Mean ± SD.

The genotoxic effect of IFNα2–Tα1 and IFNα2 in HepG2 cells and MDA-MB-231 was determined by comet assay. The slides of the comet assay were stained with EtBr and analyzed under a fluorescent microscope to observe the morphology of comets. Cometscore 2.0 software was used for the quantitative analysis of the comets. Eight parameters (comet length, comet height, head diameter, head area, percentage DNA (deoxyribonucleic acid) in head, tail length, tail area and percentage DNA in tail) were analyzed for each comet on each slide and the average of values was taken. In the structure of comets, the undamaged DNA nucleoid is referred to as the “head” and the streak of DNA that trails the nucleoid is referred as the “tail”. Analysis of the parameters revealed that in IFNα2–Tα1 fusion protein-treated HepG2 and MDA MB-231 cells, the comets had less head diameter, head area and percentage head DNA and more tail length, tail area and percentage tail DNA compared to comets formed for IFNα2-treated and control cells (Figure 4A,B). The results clearly indicated that for both cell lines, DNA damage was increased in fusion protein-treated cells.

### 3.4. Higher Antiviral Effect of IFNα2–Tα1 in HCV-Infected Huh7 Cells

**Figure 5:**
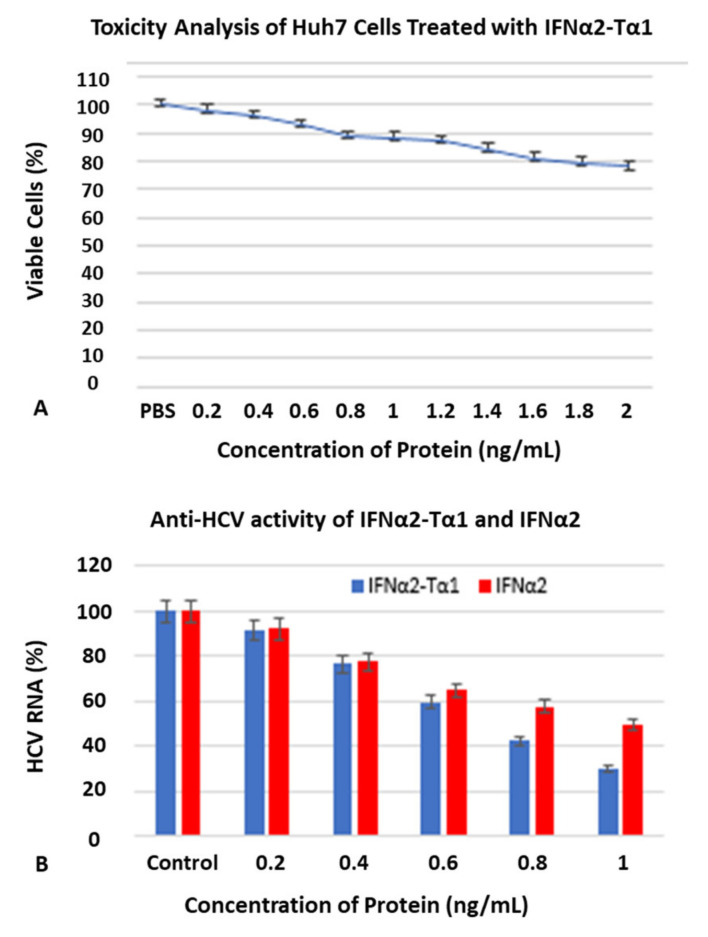
(**A**) Toxicity analysis of IFNα2–Tα1 by trypan blue exclusion test on Huh7 cells. IFNα2–Tα1 up to concentration of 2 ng/mL at which ~85% cells are viable was considered as non-toxic for cells. (**B**) IFNα2–Tα1 analyzed for anti-HCV activity in comparison with IFNα2. IFNα2–Tα1 showed higher anti-HCV activity (70.40%) in HCV-infected Huh7 cells as compared to IFNα2 (51.39%) at similar concentration. Data are represented as Mean ± SD (n = 3, two-way ANOVA, *p* < 0.05 considered significant with 95% confidence).

Huh7 cells were treated with various concentrations of IFNα2–Tα1 for toxicity analysis of the fusion protein by trypan blue exclusion test. According to our results, the concentration of IFNα2–Tα1 up to 2 ng/mL was found to be non-toxic for Huh7 cells (Figure 5A). The antiviral effect of IFNα2–Tα1 was determined by treating HCV-infected Huh7 cells with different concentrations (0.2–1 ng/mL) of IFNα2–Tα1 or reference IFNα2 for 24 h. When HCV RNA was quantified in treated cells and compared with the control cells (untreated HCV-infected cells) by quantitative real-time PCR, it was observed that HCV RNA was reduced equal to 70.40% (EC50 0.7 ng/mL) in IFNα2–Tα1-treated cells at a concentration of 1 ng/mL as compared to a 51.39% (EC50 0.99 ng/mL) reduction in IFNα2-treated cells at the same concentration (Figure 5B). The level of HCV ribonucleic acid (HCV RNA) in infected cells after treatment with IFNα2–Tα1 and reference IFNα2 is given as a percentage relative to the level of HCV RNA in control cells (Figure 5B).

### 3.5. The Relative Expression of Genes Associated with Anticancer, Antiviral and Immune-modulatory Activities in IFNα2–Tα1 and IFN-α-2 Treated Cells

**Figure 6:**
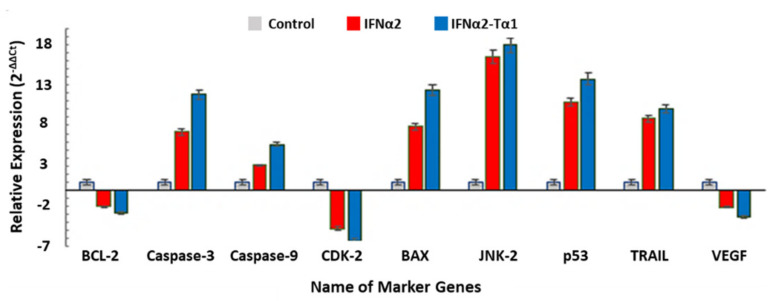
Relative expression of BCL2, CASP3, CASP9, CDK2 BAX, JNK-2, P53, TRAIL, VEGF genes measured by quantitative real-time PCR in IFNα2–Tα1-treated cells compared with IFNα2-treated cells. These data are presented as means of triplicate PCR experiments. Data are represented as Mean ± SD (n = 3, two-way ANOVA, *p* < 0.05 considered significant with 95% confidence).

**Figure 7:**
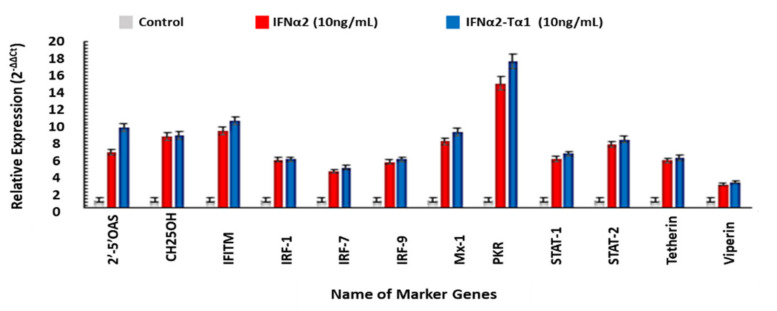
Relative expression of 2′,5′-OAS, CH25OH, IFITM, IRF-1, IRF-7, IRF-9, MX-1, PKR, STAT-1, STAT-2, Tetherin, and Viperin genes measured by quantitative real-time PCR in IFNα2–Tα1-treated cells compared with IFNα2-treated cells. These data are presented as means of triplicate PCR experiments. Data are represented as Mean ± SD (n = 3, two-way ANOVA, *p* < 0.05 considered significant with 95% confidence).

**Figure 8:**
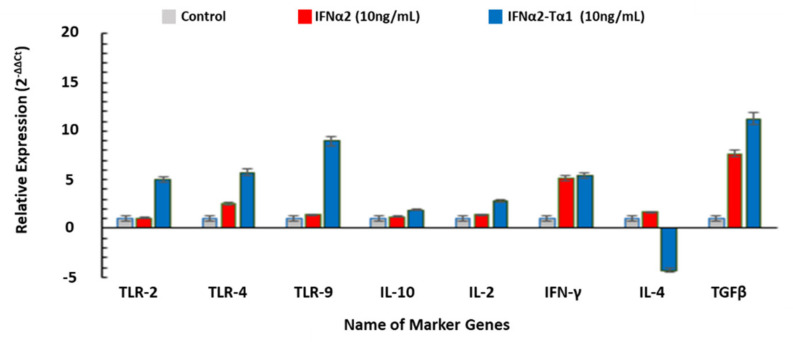
Relative expression of TLR-2, -4, -9 and other immune modulators such as IL-10, IL-2 IFN-γ, IL-4 and TGF-β in IFNα2–Tα1 and IFNα2-treated cells. These data are presented as means of triplicate PCR experiments. Data are represented as Mean ± SD (n = 3, two-way ANOVA, *p* < 0.05 considered significant with 95% confidence).

The change in the expression of genes related to anticancer, antiviral and immunomodulatory activities in IFNα2–Tα1 and IFN-α-2-treated cells was quantified by quantitative real time PCR and compared with control cells. According to our results, the expression of proapoptotic genes such as CASP3, CASP9, BAX, JNK-2, p53 and TRAIL (TNF-related apoptosis-inducing ligand) was significantly enhanced in IFNα2–Tα1-treated cells compared to IFNα2-treated cells, and the expression of anti-apoptotic genes BCL-2, CDK-2 and VEGF was lowered in the IFNα2–Tα1-treated cells compared to IFNα2-treated cells (Figure 6). It was also observed that the expression of antiviral genes 2′,5′-OAS, CH25OH, IFITM, IRF-1, IRF-7, IRF-9, Mx1, PKR, STAT-1, STAT-2, tetherin and viperin was significantly elevated in IFNα2–Tα1-treated cells as compared to IFNα2-treated cells, as shown in Figure 7. In addition, the expression of TLR-2, -4, -9 and other immune modulators such as IL-10, IL-2, IFN-γ, and TGF-β was also enhanced in IFNα2–Tα1-treated cells compared to IFNα2-treated cells, while the expression of anti-apoptotic cytokine gene IL-4 was decreased in fusion protein-treated cells as compared to IFNα2-treated cells, as shown in Figure 8.

## 4. Discussion

IFNα2 and Tα1 are used effectively for the treatment of viral infections and different types of cancers. Studies show that the use of IFNα2 and Tα1 in combination is more effective and less toxic in comparison to when both are used individually for the treatment of cancer and hepatitis [17,23,26,33,38]. These findings show that IFNα2 and Tα1 coordinate with each other to perform their activities and show synergy in activity. In our previous study, we reported the high yield production of recombinant IFNα2–Tα1 comprising IFNα2 and Tα1 for use as a substitute of IFNα2 and Tα1, which are used in combination for hepatitis and cancer therapy. We observed that recombinant IFNα2–Tα1 produced in our laboratory is biologically active and exhibits both IFNα2 and Tα1 activities [39]. In this study, we further determined the anticancer and antiviral activities of recombinant IFNα2–Tα1 in comparison with reference IFNα2 alone.

Initially, cell attachment assay was performed to determine whether the fusion of Tα1 to IFNα2 affects its binding to the tumor cells or not. We observed that IFNα2–Tα1 attaches to HepG2 cells more effectively as compared to IFNα2 alone (Figure 1A,B). These results of the cell attachment assay may help IFNα2–Tα1 to give a higher anti-proliferative effect on cells as compared to IFNα2 alone. When IFNα2–Tα1 was analyzed for its ability to inhibit the proliferation of cancer cells, it was observed that IFNα2–Tα1 inhibits the proliferation of HepG2 (Figure 2A,B) and MDA-MB-231 (Figure 3A,B) cells more effectively as compared to reference IFNα2 alone. Our result is supported by other studies in which fusion proteins produced by the genetic fusion of two genes show elevated biological effect [34,40,41,42]. In addition to anti-proliferative effect, the genotoxic effect of IFNα2–Tα1 was observed by comet assay in IFNα2-Tα fusion protein and IFNα2-treated HepG2 and MDA-MB-231 cells. Analysis of the multiple parameters of selected comets revealed that in IFNα2–Tα1-treated HepG2 and MDA MB-231 cells, the comets had less head diameter, head area and percentage head DNA and more tail length, tail area and percentage tail DNA than in IFNα2-treated and control cells (Figure 4A,B). Our results clearly indicated that the DNA damage was increased in IFNα2–Tα1-treated HepG2 and MDA-MB231 cells as reported in another study that the apoptotic cells can give comet images [43]. 

To further validate the enhanced anti-proliferative effect of IFNα2–Tα1, the expression of anticancer genes was determined in IFNα2–Tα1-treated HepG2 cells compared to IFNα2-treated cells and reported in this study for the first time. According to our observations, the expression of proapoptotic genes such as CASP3, CASP9, BAX, JNK-2, p53, and TRAIL was increased by 11.83, 5.64, 12.39, 17.93, 13.75-10.06-fold in IFNα2–Tα1-treated cells as compared to a 7.19, 3.05, 7.88, 16.52, 10.90-8.85-fold increase with IFNα2, respectively, at a similar concentration (Figure 6). At the same time, the expression of BCL-2, CDK-2 and VEGF in IFNα2–Tα1-treated cells was decreased by −2.85, −6.26 and −3.22-fold as compared to a −2.0, −4.76 and −2.08-fold decrease in cells treated with reference IFNα2, respectively, at a similar concentration (Figure 6). Our results are supported by past studies according to which the elevated expression of caspases, PKR and p53 induces apoptosis in tumorous cells, while the decreased expression of CDKs and VEGF induces cell cycle arrest at the G1 phase to give anti-proliferative effect and inhibit angiogenesis in cancerous cells, respectively. The activation of proapoptotic proteins Bax and TRAIL and the inhibition of anti-apoptotic protein (Bcl-2) also initiate apoptosis through the activation of caspases [44,45,46,47,48,49,50,51].

To explore the antiviral activity of IFNα2–Tα1, an in vitro anti-HCV assay was performed in HCV-infected Huh7 cells. First of all, it was observed by trypan blue exclusion assay that the concentration of IFNα2–Tα1 equal to 2 ng/mL is non-toxic for Huh7 cells (Figure 5A). Further, the real time PCR results showed that the HCV RNA level was reduced equal to 70.40% (EC50 0.70 ng/mL) at 1 ng/mL IFNα2–Tα1s in HCV-infected Huh7 cells as compared to a 51.39% reduction (EC50 0.99 ng/mL) with IFNα2 at a similar concentration (Figure 5B). It was observed that the EC50 of IFNα2–Tα1 for HCV replication inhibition in Huh7 cells was 0.7 ng/mL in comparison with 1 ng/mL, 18 ng/mL and 15 ng/mL for PEG-IFNα-2b, PEG-IFNα-2a and alb-IFN, respectively, in other studies [52]. 

As it is reported, IFNα2 induces the expression of various genes which are known as interferon stimulated genes (ISGs), and products of these ISGs show enhanced antiviral effect. To explore the effect of IFNα2–Tα1 on the expression of ISGs, the expression of different ISGs was quantified in cells treated with IFNα2–Tα1 and compared with expression in IFNα2-treated cells. Our results showed that the expression of genes such as 2′,5′-OAS, IRF-1, IRF-7, IRF-9, PKR, STAT-1-STAT-2-CH25OH, IFITM, Mx-1, tetherin and viperin was increased by 9.72, 5.91, 4.91, 5.91, 17.72, 6.56, 8.27–8.76, 10.54, 9.17, 6.05 and 3.17-fold in IFNα2–Tα1-treated cells as compared to a 6.71, 5.84, 4.44, 5.57, 15.06, 5.97, 7.70–8.65, 9.27, 8.07, 5.78–2.85-fold increase in cells treated with reference IFNα2 alone at the same concentration, respectively (Figure 7). The expression of these genes by IFNα2–Tα1 is reported for the first time in this study, but the elevated expression of ISGs such as 2′,5′-OAS IRF-1, IRF-7, IRF-9, PKR, STAT-1, STAT-2 and Mx-1 by IFNα2 is already reported in other studies [1,7,53,54]. 

Furthermore, the expression of genes encoding TLR-2, TLR-4, and TLR-9 was elevated by 5.03, 11.56, and 9.83-fold in IFNα2–Tα1-treated cells as compared to a 2.21, 6.55, and 6.11-fold change by reference IFNα2 alone at the same concentration (Figure 8). According to other studies, the up-regulated expression of TLRs provides defense against viral infections through the TLR signaling pathway [55,56,57], and this pathway acts as a first line of antiviral immunity [58].

Lastly, the expression of genes encoding proteins with immunomodulatory effects was determined by quantitative real-time PCR as it is reported in the literature that both IFNα2 and Tα1 also modulate immune response to inhibit virus replication and suppress the growth of cancerous cells. Our results showed that the level of IL-10, IFN-γ, IL2 and TGF-β was increased in both IFNα2–Tα1 and IFNα2-treated cells, but in the case of cells treated with IFNα2–Tα1, the level of IL-10, IFN-γ, IL2 and TGF-β was increased by 1.90, 5.39, 2.82–11.29-fold as compared to a 1.21, 5.20, 1.39–7.70-fold increase in cells treated with reference IFNα2 alone at the same concentration, respectively (Figure 8). Our results also corroborate other studies according to which the activation of cytokines such as IL-2 and IFN-γ inhibits HCV infection and gives higher anticancer effect [23,38,48,59]. The activated TGF-β induces an immunoregulatory effect to promote apoptosis and give an anti-proliferative effect [60]. The activation of anti-inflammatory cytokine (IL-10) by IFNα2–Tα1 further supports the therapeutic use of IFNα2–Tα1 without damage to host cells [61]. Interestingly, the level of IL-4 was decreased by -4.28-fold in IFNα2–Tα1-treated cells as compared to a 1.67-fold increase in cells treated with reference IFNα2 (Figure 8), respectively. It is also reported that IL-4 activates the growth of tumor cells through the inhibition of apoptosis. The decreased level of IL-4 expression in cells treated with IFNα2–Tα1s might support the apoptosis [62].

## 5. Conclusions

This study concludes that recombinant IFNα2–Tα1 exhibits elevated anticancer and antiviral activities as compared to IFNα2 alone. The results of anticancer and antiviral assays as well as the change in expression of genes encoding effector proteins related to anticancer and antiviral activities in IFNα2–Tα1-treated cells compared to IFNα2-treated cells support our hypothesis. This enhanced effectiveness of IFNα2–Tα1 might be due to the coordinated and synergistic effect of IFNα2 and Tα1 in IFNα2–Tα1. In the future, IFNα2–Tα1 further needs in vivo trials to explore pathways by which it showed higher antiviral and anticancer activities.

## Abbreviations

NK	Natural killer cells
DCs	Dendritic cells
TLR	Toll-like receptor
2′,5′-OAS	2′,5′-oligoadenylate
IRF	Interferon regulatory factor
STAT	Signal Transducer and Activator of Transcription
PKR	Protein Kinase R
JNK-2	c-Jun N-terminal kinase-2
IFITM	Interferon-inducible transmembrane protein
CH_25_OH	Cholesterol-25-hydroxylase
BCL-2	B-cell lymphoma 2
BAX	BCL2 associated X
CDK	Cyclin-dependent kinases
Mx-1	Myxovirus resistance-1
TGF-β	Transforming growth factor-beta
TRAIL	TNF-related apoptosis-inducing ligand
CASP	Caspase
